# Applying mathematical modelling to estimate the impact of COVID-19-related VMMC service disruptions on new HIV infections in Zimbabwe

**DOI:** 10.1186/s12879-023-08081-7

**Published:** 2023-02-23

**Authors:** Newton Chagoma, Tiwonge Kanyenda, Bothwell Pindiwe, Howard Nyika, Lawrence Nyazema, John Stover, Danielle Resar, Natsai Shoko, Sarah Jenkins, Yemurai Katanda, Sinokuthemba Xaba, Owen Mugurungi

**Affiliations:** 1 Clinton Health Access Initiative, Boston, USA; 2 Clinton Health Access Initiative, Harare, Zimbabwe; 3 Ministry of Health and Child Care, Harare, Zimbabwe; 4 Avenir Health, Glastonbury, USA

**Keywords:** VMMC, HIV Prevention, Modelling, COVID-19 impact

## Abstract

**Background:**

The COVID-19 pandemic has overwhelmed health systems with knock on effects on diagnosis, treatment, and care. To mitigate the impact, the government of Zimbabwe enforced a strict lockdown beginning 30 March 2020 which ran intermittently until early 2021. In this period, the Ministry of Health and Childcare strategically prioritized delivery of services leading to partial and full suspension of services considered non-essential, including HIV prevention. As a result, Voluntary Medical Male Circumcision (VMMC) services were disrupted leading to an 80% decline in circumcisions conducted in 2020. Given the efficacy of VMMC, we quantified the potential effects of VMMC service disruption on new HIV infections in Zimbabwe.

**Methods:**

We applied the GOALS model to evaluate the impact of COVID-19-related disruptions on reducing new HIV infections over 30-years. GOALS is an HIV simulation model that estimates number of new HIV infections based on sexual behaviours of population groups. The model is parameterized based on national surveys and HIV program data. We hypothesized three coverage scenarios by 2030: scenario I - pre-COVID trajectory: 80% VMMC coverage; Scenario II - marginal COVID-19 impact: 60% VMMC coverage, and scenario III - severe COVID-19 impact: 45% VMMC coverage. VMMC coverage between 2020 and 2030 was linearly interpolated to attain the estimated coverage and then held constant from 2030 to 2050, and discounted outcomes at 3%.

**Results:**

Compared to the baseline scenario I, in scenario II, we estimated that the disruption of VMMC services would generate an average of 200 (176–224) additional new infections per year and 7,200 new HIV infections over the next 30 years. For scenario III, we estimated an average of 413 (389–437) additional new HIV infections per year and 15,000 new HIV infections over the next 30 years. The disruption of VMMC services could generate additional future HIV treatment costs ranging from $27 million to $55 million dollars across scenarios II and III, respectively.

**Conclusion:**

COVID-19 disruptions destabilized delivery of VMMC services which could contribute to an additional 7,200 new infections over the next 30 years. Unless mitigated, these disruptions could derail the national goals of reducing new infections by 2030.

## Background

The COVID-19 pandemic has exerted enormous pressure on health systems in Southern Africa, and across the globe. The pandemic itself, in addition to measures to curb its spread, has decreased global life expectancy [1] and triggered serious negative impact on economic progress and social interactions. Global statistics indicate that nearly 430 million cases of COVID-19 have been diagnosed, with over 5.8 million deaths registered as of February 2022 [2]. Zimbabwe registered a cumulative total of over 233,000 confirmed cases and 3,388 deaths during that period [2]. As with many other countries, the pandemic has undermined the health system in Zimbabwe, which already faced challenges with constrained capacity and resources for routine operations, with an estimated two skilled health providers (physicians, nurses, and midwives) per 1,000 population in 2018 [3]. As COVID-19 cases spiked and hospitalizations began, the government of Zimbabwe enforced a strict lockdown beginning 30 March 2020 which ran intermittently until early 2021. To align with the national guidance, the Ministry of Health and Childcare (MOHCC) strategically prioritized delivery of health services, leading to partial and full suspension of services that were deemed non-essential, including HIV prevention services.

**Fig. 1:**
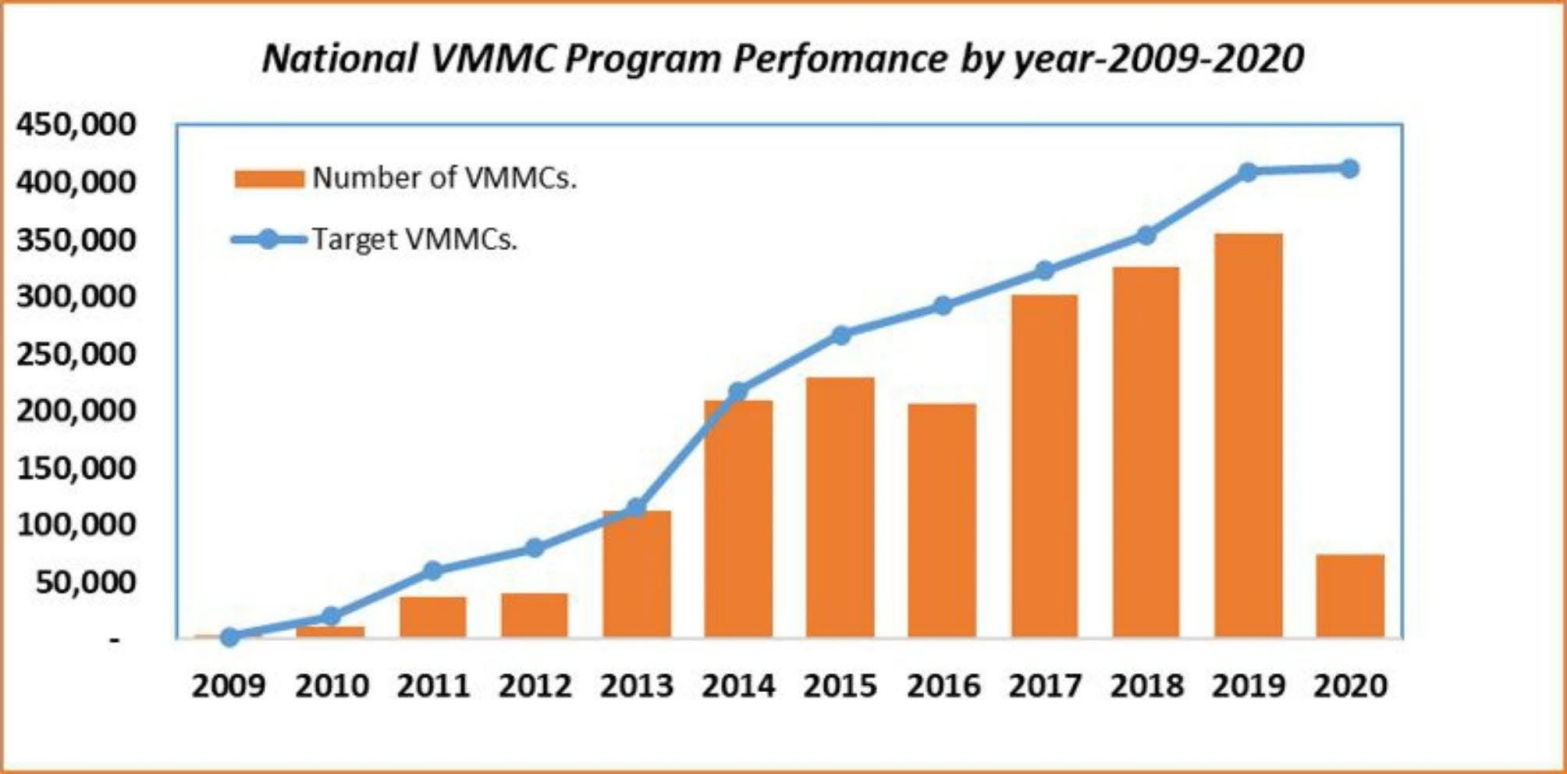
Historical Performance Trend of VMMC Program. Source: Authors’ own based on VMMC program reports; year implies Jan-Dec

Voluntary Medical Male Circumcision (VMMC) is an effective HIV prevention intervention, reducing the lifetime risk of heterosexual HIV transmission by approximately 60% [4]. Despite its critical role in reducing the risk of HIV acquisition, VMMC was one of the health services that was indefinitely suspended to mitigate the spread of COVID-19 [5]. COVID-19 restrictions further resulted in significant changes to VMMC service delivery and demand generation models which had been predominantly conducted through in-person outreach. Consequently, the number of men circumcised in 2020 decreased by 80% compared to 2019 (Fig. 1). While demand generation models have been adapted during the pandemic to leverage virtual platforms, these disruptions are likely to be prolonged until COVID-19 vaccines are sufficiently scaled up or community transmission within Zimbabwe significantly decreases.

In Zimbabwe, in 2019, annual incidence of HIV among adults (ages 15 years and older) was approximately 31,000 new cases [6]. As Zimbabwe strives to achieve the 2030 goal of reducing new HIV infections by 90%, VMMC will remain a core intervention in the package of prevention services available to men. However, the COVID-19-related disruption of services presents a threat to achieving the full potential of VMMC in reducing HIV incidence. In this analysis, we sought to quantify the impact of COVID-19 related disruptions of VMMC services on the number of new HIV infections in Zimbabwe by 2030, including the additional HIV treatment costs.

## Methods

### Study design

This modelling study applied the generic Goals HIV mathematical model to evaluate the impact of COVID-19-related disruptions on the delivery of VMMC and its impact on reducing new HIV infections over a 30-year period.

### Model and data sources

Goals is an HIV simulation model that estimates number of new HIV infections based on sexual behaviours of population groups assigned to different risk groups and their subsequent sexual mixing. A person enters the model at age 15 and the model distributes the populations by risk groups categorized as: men and women with non-regular partners, men and women with only one regular partner, men who have sex with men, and sex workers and their clients. The underlying behavioural information from each group is utilized to estimate the probability of HIV transmission. Biomedical interventions such as VMMC can reduce the risk of HIV acquisition as demonstrated in several randomized control trials [7].

Following Weinstein [8], the basic equation of the probability of HIV transmission is structured as follows:

$${Prob}_{g}=1-[ {Prev}_{{g}^{{\prime }}}* {\left(1-r\right)}^{acts }+{(1-Prev}_{{g}^{{\prime }}}){]}^{partners}$$

where, “r” is probability of HIV transmission per act;

“g” is risk group;

“acts” represents the number of sexual acts per partner per year;

“partners” represents the number of different partners per year;

“prev” is the HIV prevalence in the partner population; and.

“prob” is the probability of acquiring HIV infection per year.

To quantify the impact of HIV interventions, including VMMC on HIV prevention, the following scope of data are thus needed [9]:

Demographic data—size of adult population disaggregated by sex and age, adult population growth rate, crude birth rate, crude death rate, proportion of population alive at 15 years (age of sex debut) and historical male circumcision prevalence.

Epidemiologic data—HIV prevalence in adult population (15–49 age bracket) disaggregated by sex and age; efficacy of VMMC; risk factors for HIV transmission factors based on published literature.

Sexual behaviors data—Sexual mixing patterns, sexual acts, number of sexual partners and condom use.

The required data values were extracted from nationally representative population-based surveys such as Demographic Health Surveys (DHS), Public Health Impact Assessments (PHIA), and Integrated Behavioral and Biological Surveys (IBBS) targeting key populations, published literature, and available program reports. In the model, the probability of HIV transmission is captured as a function of HIV prevalence and the underlying force of infection based on changes in sexual behaviors (sexual mixing). As Goals is a sexual behavior model, only circumcisions in sexually active males are accounted for in the transmission process. Sexual activity is assumed to start at age 15, and existing evidence suggest a rising trend in VMMC prevalence in the 15–49 age brackets estimated as 10.3% in 2005 and 9% in 2010 based on the DHS. The PHIA reported VMMC coverage of 14% in 2016 while the Decision Makers Program Planning Tool (DMPPT) estimated national coverage of 25% in 2019. Avenir Health fitted and calibrated the model based on these parameter values to reflect the historical pattern of the pandemic. Together with HIV incidence and prevalence, these values were transferred from the Goals to the Spectrum’s AIDS Impact Model (AIM) (linked modules) to quantify and retrieve model outputs based on the hypothesized scenarios. Relevant outputs were aligned to the objectives of the model and included the number of new HIV infections, mortality, and the number of people living with HIV, among others.

The Goals model was developed by Avenir Health and is publicly available. Full details of the model can be found in the appendix [10]. The model is part of the Spectrum software which can be downloaded at www.avenirhealth.org.

### Analytical approach

To quantify the impact of COVID-19 disruptions on new HIV infections, we hypothesized three VMMC coverage scenarios described below.

**Scenario I**: Pre-COVID trajectory (baseline): we assumed that coverage of VMMC would steadily increase at pre-COVID-19 levels and the country would achieve 80% population coverage in the 15–49-year age group by 2030 as outlined in the Sustainability Transition Implementation Plan-STIP 2019–2021 [11].

**Scenario II**: Marginal COVID-19 impact: we assumed limited impact of disruptions on VMMC service delivery resulting in 60% VMMC coverage in the 15-49-year age group by 2030. This scenario was informed by evidence from a multi-country study, including Zimbabwe, suggesting minimal impact on service coverage [12] and the observed comparable uptake of services before, during, and after COVID-19 restrictions.

**Scenario III**: Severe COVID-19 impact: we assumed that potential volatility in COVID-19 infections and restrictions and uncertainty about the timeline for stabilization of health service to pre-COVID-19 levels would drastically disrupt provision and uptake of VMMC services. We hypothesized VMMC coverage of 45% in the 15–49-year age group by 2030.

In all the three scenarios, we used historical VMMC data for 2014–2019. VMMC coverage between 2020 and 2030 was linearly interpolated to attain the estimated coverage for each scenario and then held constant from 2030 to 2050.

Given that the benefits of VMMC accrue over time, the time horizon for the model was extended to 30 years to allow the model to fully estimate the impact of disruptions. Discounting of outcomes was done at 3% in line with regional recommendations [13]. Based on evidence from VMMC and Antiretroviral Therapy (ART) program implementation, the cost of VMMC was estimated at $90 per procedure in 2018. The fully loaded cost of ART was estimated at $165 per person per year. The “fully-loaded” cost includes the cost of drugs, laboratory tests, human resources, and other items relevant in the provision of ART services [14]. As HIV requires life-long treatment with ART, we also estimated the discounted life-time cost of ART based on the fully loaded cost of ART. The discounted life-time cost of first-line ART was estimated around $3,710 per person assuming average duration on treatment of 33 years and constant annual treatment cost discounted at 3% per year. The impact of COVID-19 disruptions on Zimbabwe’s VMMC program was estimated as the difference between the modelled new HIV infections in the scenario I (baseline) and the modelled new HIV infections in scenarios II and III. The additional future treatment costs were estimated as the multiplicative function of additional new HIV infections per scenario and the discounted life-time cost of ART.

## Results

Compared to the baseline scenario I, in scenario II (60% target population coverage), we estimated that the disruption of VMMC services would generate an average of 200 (176–224) additional new infections per year and 7,200 new HIV infections over the next 30 years. Similarly, for scenario III (45% target population coverage), we estimated an average of 413 (389–437) additional new HIV infections per year and 15,000 new HIV infections over the next 30 years. Key findings on the estimated effects of COVID-19-driven disruption of VMMC delivery, including gross future HIV treatment costs are summarized in Table 1.

**Table 1:** Summary trends of average new HIV infections and program costs attributable to COVID-related disruptions

Description	Scenario II (60% coverage)	Scenario III (45% coverage)
Average additional new HIV Infections per year	200 [176–224]	413 [389–437]
Average life-time treatment cost per person ($)	3,710	3,710
Cumulative total additional new HIV infections by 2030	7,212	14,865
Total Annual Life-time HIV Treatment Cost ($)	26,756,520	55,149,150

The disruption of VMMC services could generate additional future HIV treatment costs ranging from $27 million to $55 million dollars across scenarios II and III, respectively. The analysis did not account for the lower VMMC costs in scenarios II and III, the costs of second-line ART, and treatment and management of HIV-related opportunistic infections. Cost estimates would rise further if either the societal or patients’ perspective were considered [13] in this analysis. Overall future HIV treatment program costs could be higher than estimated in this analysis.

## Discussion

**Fig. 2:**
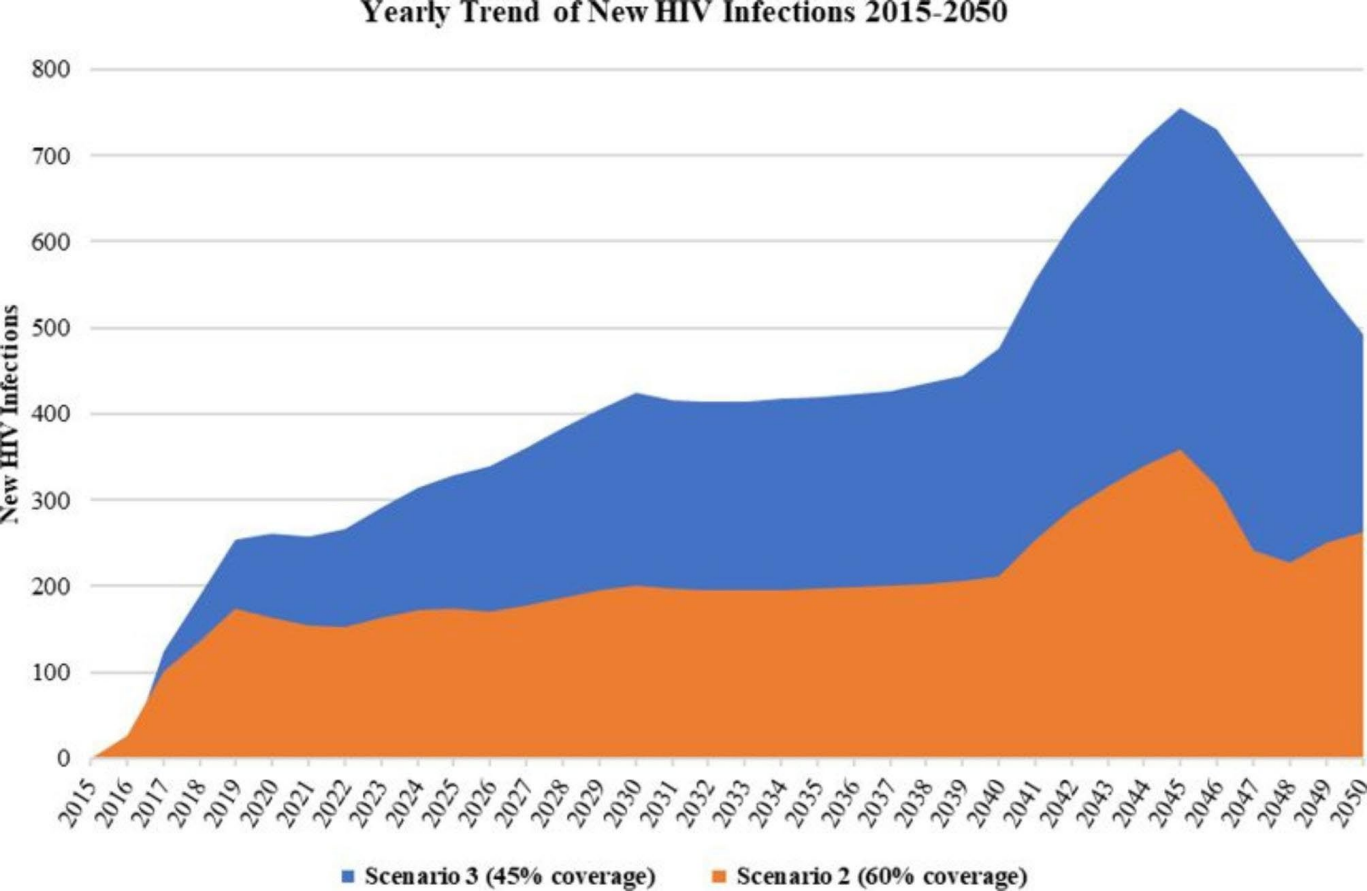
Projected Annual New HIV Infections

In Zimbabwe, COVID-19 disruptions destabilized delivery of numerous services, including VMMC, leading to an 80% reduction of VMMC procedures in 2020 compared to 2019. If disruptions persist, the VMMC program is unlikely to attain 80% coverage of services by 2030 in the 15–29 priority age group as stipulated in Sustainability and Transition Plan (2019–2021). Understanding the magnitude of VMMC service disruptions on new HIV infections (Fig. 2 below), including potential costs, may inform development and operationalization of innovative policies to accelerate uptake of services in the post-pandemic era.

Sufficient coverage of COVID-19 vaccines and subsequent reduction of COVID-19 cases, particularly cases of community transmission, will likely signal resumption of VMMC services. While the country has approved use of five COVID-19 vaccines (Sinopharm, Sinovac, Sputnik V, Covaxin and Johnson & Johnson), population coverage remains low at an estimated 7.8 million vaccine doses administered across a population of over 14 million in February 2022 [15]. Emerging strains of coronavirus disease further threaten the full resumption of VMMC and other services.

The findings presented here differ slightly from two recent modelling studies [12, 16]. In these studies, authors concluded that the COVID-19 response in sub-Saharan Africa only had a transitory effect on new HIV infections. The differences in findings between these two studies and the modelling exercise presented here can be attributed to the differences in the time horizons considered. For example, Jewell [16] and Stover’s [12] models assumed a short-term disruption (3-months, 6-months, 2-years) followed by a return to previous VMMC levels while our model assumes the impact of disruptions is extended over 30 years. Given the life-long impact of impact of VMMC it is important to consider a longer-time frame for analysis to account for the transition of younger people into peak periods of sexual activity. Evidence from modelling studies assuming short term disruptions may have contributed to UNAIDS guidance on temporary suspension of VMMC services during the COVID-19 pandemic [17]. However, the results of this longer-term VMMC-specific model provides an important perspective to weighing the costs and benefits of public health responses in the context of the HIV epidemic.

The return of VMMC services to pre-COVID-19 levels will likely require implementation of deliberate policies and practices to catalyze uptake of services based on country context. In Zimbabwe, models leveraging mobile service delivery and outreach-focused demand generation were severely obstructed by COVID-19 pandemic restrictions on gathering in groups. Evidence from Zambia suggests that routinization and static delivery of VMMC services can contribute to program continuation and performance (CHAI, 2021). Adapting service delivery and demand generation models for VMMC and other HIV prevention programs to respond to emerging threats will facilitate continued progress towards achieving epidemic control. Further, global investments in health systems strengthening will also be critical to enhancing resilience of health systems and minimizing the disruption of VMMC and other health services. While contexts may differ, cross-country sharing of program learnings targeting policy makers and program managers could inform operationalization of effective and strategic policies that can sustain service delivery through pandemics and other national and global crises.

Further research should consider comparing the population health impact of COVID-19 to the long-term impact of VMMC service disruptions on HIV incidence estimated in this study to inform program decision-making. This study did not estimate the impact of service disruption or reduced access to other HIV prevention interventions such as condoms and oral pre-exposure prophylaxis on averting future HIV infections. Further research could evaluate the holistic impact of disruption of all HIV prevention services on future HIV infections and lifetime treatment costs.

## Limitations

This study has several important limitations. First, a predictive model is only as strong as the underlying data. The data inputs that informed model development and calibration such as HIV incidence, prevalence, and VMMC coverage were extracted from national surveys such as DHS and PHIA. These survey findings are subject to inherent bias because they include sensitive questions around respondents’ HIV serostatus and circumcision status. Second, the generic Goals model applies a single age bracket 15–49 to determine incidence of new infections. Compared to the Goals-ASM and DMPPT models which require age-disaggregated input parameters for coverage of services, the quantified additional new infections observed in our analysis could differ due to variable HIV risk by age group. For example, applying a single age bracket incidence rate may result in underestimating additional new infections if disrupted services disproportionately impact an age segment with a higher HIV incidence.

Another major limitation to this study is the potential of confounding due to inability to control for other policy instruments in the modelling. Particularly, assumptions for Scenario II - which are informed by observed differences in service uptake prior to and during COVID-19. One such variable is the 2020 WHO recommendation to restrict delivery of circumcision services in individuals less than 15 years of age due to lack of full-informed consent on a life-long procedure as well as concerns around adverse events. Thus, the observed decrease in outputs seen in 2020, which informed Scenario II, may be partly attributed to global guidance on VMMC programming. However, the impact of this global guidance is unlikely to have been the main driver in the decrease of VMMCs performed in Zimbabwe, as the proportions of VMMCs performed in the below 15 age group remained high, accounting for about a third of total VMMCs in 2020 compared to just over 40% in 2019 [18]. Given these limitations, the extent of disruption and predicted new HIV infections discussed in this study should be considered as estimates and compared to real-life trends to assess model accuracy.

## Conclusion

HIV and COVID-19 require different treatments and programmatic strategies; however, disease prevention and mitigation efforts do not exist in siloes. Strategies to mitigate COVID-19 have had an impact on many public health outcomes, including for HIV. The drastic reduction in the availability of VMMC and other HIV prevention services will contribute to additional new HIV infections depending on the length and frequency of disruptions. Unless these disruptions are effectively mitigated, they could reverse decades of sustained progress in reducing new HIV infections and achieving epidemic control. It is important that decision makers consider strategic investments and policies to support COVID-19 pandemic control, while maintaining and sustaining delivery of preventive health services such as VMMC. Modelling will continue to be a valuable tool to inform evidence-based decision-making and to understand health system trade-offs.

## Data Availability

The datasets used and/or analysed during the current study are available from the corresponding author on reasonable request.

## List of Abbreviations

ART: Anti retroviral
DHS: Demographic Health Survey
DMPPT: Decision Makers Program Planning Tool
IBBS: Integrated Behavioural and Biological Surveys
MOHCC: Ministry of Health and Child Care
PHIA: Public Health Impact Assessment
STIP: Sustainability Transition Implementation Plan
VMMC: Voluntary Medical Male Circumcision
